# Retrospective analysis of Guillain–Barré syndrome and Fisher syndrome after the Great East Japan Earthquake

**DOI:** 10.1002/brb3.234

**Published:** 2014-04-30

**Authors:** Hirofumi Tsuboi, Naoto Sugeno, Maki Tateyama, Ichiro Nakashima, Takafumi Hasegawa, Hiroshi Kuroda, Kimihiko Kaneko, Michiko Kobayashi, Aya Ishigaki, Juichi Fujimori, Masashi Aoki

**Affiliations:** 1 Department of Neurology, Tohoku University HospitalSendai, 980-8574, Japan; 2 Department of Neurology, Tohoku Pharmaceutical University HospitalSendai, 983-0005, Japan

**Keywords:** Disaster, epidemiology, Fisher syndrome, Guillain–Barré syndrome

## Abstract

Guillain-Barré syndrome (GBS) and Fisher syndrome (FS) are immune-mediated peripheral neuropathies, and most of these cases were known to be associated with a preceding infection. Recent reports evidenced an increase in the number of infectious disease cases after the earthquake. The aim of this report is to investigate the incidence and clinical features of GBS and FS after the Great East Japan Earthquake. We found GBS and FS patients had markedly increased in 2011, the year of the earthquake. In regard to an antecedent illness, gastrointestinal infection was significantly increased in GBS patients after the earthquake. These results suggest environmental factors including infectious agents and stress caused by the earthquake might have been involved in the outbreak of the diseases.

The Great East Japan Earthquake, with the epicenter ∼70 km east of Miyagi Prefecture, occurred at 14:46 on 11 March 2011. An extensive area of the coast of this district was severely damaged by the tsunami and the number of fatalities exceeded 28,500 (Shibahara 2011). In addition, because the people suffered from the total destruction of their homes and lifelines, they had to live in unsanitary and unhealthy places for several months. Recent reports suggested an increase in the number of infectious disease cases after the earthquake (Aoyagi et al. 2013). Guillain–Barré syndrome (GBS) and Fisher syndrome (FS) are known as acute immune-mediated peripheral neuropathies, and nearly 50% of GBS and 70% of FS patients have had a preceding infection (Jacobs et al. 1998; Koga et al. 2005). Therefore, a higher incidence of GBS and FS is expected after disasters including large earthquakes, but there have been few reports concerning this issue to our knowledge. The aim of this report is to investigate the incidence and clinical features of GBS and FS after the earthquake.

All hospitalized patients diagnosed as GBS or FS from 1 March 2007 to 28 February 2013, in two major hospitals located in Sendai-city, Miyagi Prefecture, were analyzed. The area covered by these hospitals did not change after the earthquake. There were 26 patients of GBS and 10 patients of FS. The new-onset patient counts were collected from March to the following February in each year. The annual incidence of GBS and FS before the earthquake was about five patients per year, but in March 2011 to February 2012, the year just after the earthquake, such patients had markedly increased (Fig. 1A), and most of the patients were documented in the first 6 months (March to August, 2011) after the earthquake (Fig. 1B). The number of patients during this period was significantly greater than the mean score of the ordinal years 2007–2010 (*P* < 0.05). Gender, age, cerebrospinal fluid protein levels, treatments, and prognosis showed no differences after the earthquake. GBS patients with gastrointestinal infection as an antecedent illness had significantly increased to 63% in the year after the earthquake compared with before (13%, *P* < 0.05). Gastrointestinal infection has been linked to axonal GBS (Koga et al. 2005), and the electrophysiologically diagnosed axonal-type patients in this study had also increased to 50% in the year of the earthquake from 25% before the earthquake, but it was not statistically significant (*P* = 0.34). We could not test antibodies against *Campylobacter jejuni* in our patients.

**Figure 1:**
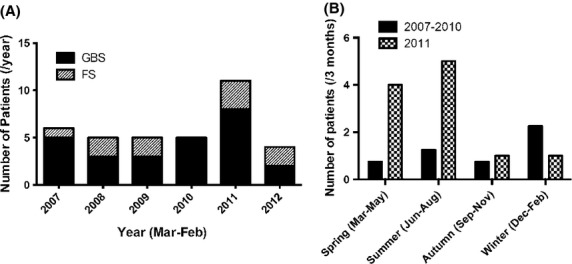
(A) Serial changes of Guillain–Barré syndrome (GBS) and Fisher syndrome (FS) patients. Counts were started from March to the following February in each year. In 2011, the year of the earthquake, such patients had markedly increased. (B) From spring (Mar–May) to summer (June–August) of 2011, the number of patients per 3-month-period was significantly greater than the seasonal mean score of the years 2007–2010 (*P* < 0.05, chi-square test), and the incidence normalized one half year after the earthquake.

According to previous reports concerning the earthquake, gastroenteritis outbreaks were sometimes prolonged after the disaster because of the continuous sanitary problems of the refuges or lifeline problems (Karmakar et al. 2008). In the present study, the higher occurrence of GBS and FS was also prolonged for several months, and gastroenteritis might have been a possible triggering factor in these disorders. In addition, noninfectious environmental factors might have played a role (Temajo and Howard 2014). After the Hanshin-Awaji earthquake, the recurrence of endogenous uveitis increased, suggesting the influence of physiological and psychological stress due to the disaster (Yamamoto et al. 1997). After the Great East Japan earthquake, psychological stress was noticed as a significant factor in inducing several internal diseases (Kanno et al. 2013; Nakamura et al. 2013). The GBS patients who were described for the first time by Guillain and Barré were the troops of World War I (Lanska 2009) under prolonged, poor hygiene with much physical and also psychological stress. In this regard, war and catastrophic disaster appear to create similar health problems.

In conclusion, GBS and FS cases increased during the several months after the earthquake and gastroenteritis was the most prevalent preceding infection in the GBS cases during this period. Although this study included only a small number of patients, the findings suggest environmental factors including infectious agents and stress caused by the earthquake might have been involved in the outbreak of the disease.
